# Atomically precise metal chalcogenide supertetrahedral clusters: frameworks to molecules, and structure to function

**DOI:** 10.1093/nsr/nwab076

**Published:** 2021-04-30

**Authors:** Jiaxu Zhang, Pingyun Feng, Xianhui Bu, Tao Wu

**Affiliations:** College of Chemistry, Chemical Engineering and Material Science, Soochow University, Suzhou 215123, China; Department of Chemistry, University of California, Riverside, CA 92521, USA; Department of Chemistry and Biochemistry, California State University, Long Beach, CA 90840, USA; College of Chemistry and Materials Science, Guangdong Provincial Key Laboratory of Functional Supramolecular Coordination Materials and Applications, Jinan University, Guangzhou 510632, China; College of Chemistry, Chemical Engineering and Material Science, Soochow University, Suzhou 215123, China

**Keywords:** metal chalcogenide, nanocluster, open framework, semiconductor, reticular chemistry, atomically precise nanochemistry

## Abstract

Metal chalcogenide supertetrahedral clusters (MCSCs) are of significance for developing crystalline porous framework materials and atomically precise cluster chemistry. Early research interest focused on the synthetic and structural chemistry of MCSC-based porous semiconductor materials with different cluster sizes/compositions and their applications in adsorption-based separation and optoelectronics. More recently, focus has shifted to the cluster chemistry of MCSCs to establish atomically precise structure–composition–property relationships, which are critical for regulating the properties and expanding the applications of MCSCs. Importantly, MCSCs are similar to II–VI or I–III–VI semiconductor nanocrystals (also called quantum dots, QDs) but avoid their inherent size polydispersity and structural ambiguity. Thus, discrete MCSCs, especially those that are solution-processable, could provide models for understanding various issues that cannot be easily clarified using QDs. This review covers three decades of efforts on MCSCs, including advancements in MCSC-based open frameworks (reticular chemistry), the precise structure–property relationships of MCSCs (cluster chemistry), and the functionalization and applications of MCSC-based microcrystals. An outlook on remaining problems to be solved and future trends is also presented.

## Teaser

The development of metal chalcogenide supertetrahedral clusters over the past three decades was concentrated in this review, ranging from the construction of cluster-based frameworks to the exploration of atomically precise cluster chemistry.

## INTRODUCTION

Nanoclusters, which consist of several or even thousands of atoms, represent an important intermediate state between microscopic atoms and macroscopic matter [1]. A profound comprehension of the composition, structure and properties of nanoclusters is crucial for exploring or extending their applications. Among the numerous types of nanoclusters, metal chalcogenide supertetrahedral clusters (MCSCs) have attracted great attention since the 1980s for their uniform sizes, well-defined structures and semiconductor properties [2,3]. Notably, because of their resemblance to II–VI or I–III–VI semiconductor nanocrystals (also known as quantum dots, QDs), MCSCs have been regarded as atomically precise ultrasmall QDs and used to clarify various issues that could not be resolved using traditional QDs, such as the determination of precise site-dependent structure–property relationships. MCSCs can be subdivided into three types: supertetrahedral T*n*-type clusters (T represents the tetrahedra while *n* indicates the metal layers in each cluster), capped supertetrahedral C*n-*type clusters (the C*n* cluster has a regular T*n* at the core covered with a shell whose stoichiometry is related to the T*n*) and penta-supertetrahedral P*n*-type clusters (the P*n* cluster is regarded as the assembly of four T*n* clusters capped onto the four faces of one anti-T*n* cluster, where an anti-T*n* has the position of cations and anions exchanged when compared with the regular T*n*) [2,3]. This review mainly focuses on ‘naked’ T*n* and P*n* clusters consisting of transition metals(M(I/II)) and group 13/14/16 elements. As C*n* and P*n* clusters with covalently capped ligands have been covered in previous reviews [4,5], only significant advances and some unique cases are presented in this review.

Generally, research on MCSCs can be categorized into three topics: (1) expansion of the architecture of crystalline MCSC-based frameworks through fabricating clusters with different sizes/compositions and modifying intercluster connecting modes, (2) discretization of MCSCs in the lattice and their solution processability and (3) exploration of the composition–structure–property relationships, functionality and applications of MCSC-based crystals. Our previous review in 2005 introduced the origin of MCSCs (1989–2005) and the development of topic 1 [2], while the review in 2020 mainly covered the progress of topics 2 and 3 (2005–2020) [3]. Based on these topics, this review provides a systematic overview of the development of MCSCs over the past three decades, with a focus on four aspects: (1) development of MCSCs of various types and sizes, (2) construction of MCSC-based open frameworks, (3) discretization and dispersibility of MCSCs and (4) site-dependent properties and applications of MCSC-based materials.

## DEVELOPMENT OF MCSCS: TYPES AND SIZES

The mimicking of natural minerals gave rise to artificial zeolites, and such crystalline porous materials have received considerable attention since the late 1940s [6]. Initially, porous materials were overwhelmingly dominated by oxide or mixed oxide/fluoride/phosphate matrices. The insulating properties of oxide zeolites and zeolite type materials have seriously restricted the development of photoelectricity applications. Metal chalcogenide zeolite type materials, which integrate semiconductivity and porosity, can overcome these limitations. Unlike the TO_4_ (T = Si^4+^ or Al^3+^) primary building units of oxide zeolites [7], metal chalcogenide supertetrahedral clusters (MCSC) with tetrahedrally coordinated metal cations and chalcogenide anions serve as the secondary building units in metal chalcogenide frameworks [8]. This section mainly focuses on MCSCs by treating them as virtual isolated clusters, whereas MCSC-based open frameworks are discussed in the next section.

**Figure 1.**
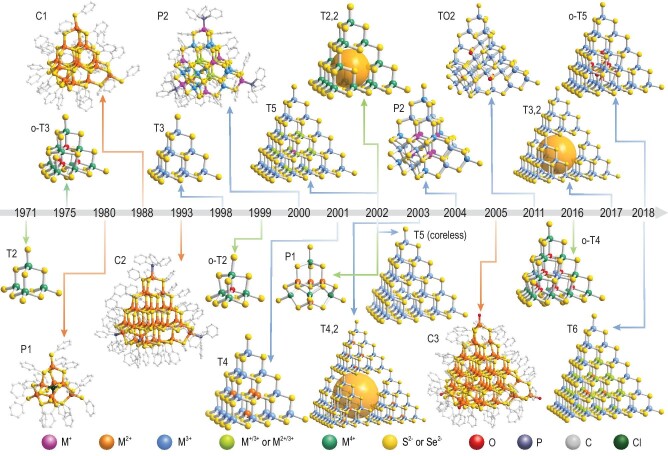
Development of the metal chalcogenide supertetrahedral cluster (MCSC) family, including basic supertetrahedral T*n*-type clusters, capped supertetrahedral C*n-*type clusters, penta-supertetrahedral P*n*-type clusters, pseudo-T*n* clusters (oxychalcogenide o-T*n* clusters, super-supertetrahedral T*p, q* clusters and coreless T*n* clusters) and TO2 clusters. Note that the MCSCs illustrated here are the first reported cases corresponding to years when they were reported: T2 [9], T3 [10], T4 [11], T5 (coreless) [27], T5 [12], T6 [13], o-T2 [22], o-T3 [23], o-T4 [24], o-T5 [25], T2,2 [30], T4,2 [31], T3,2 [32], C1 [35], C2 [36], C3 [34], covalently protected P1 [40], naked P1 [42], covalently protected P2 [49], naked P2 [46], TO2 [26].

The first series of MCSCs is supertetrahedral T*n*-type clusters with the molecular formula M*_x_*E*_y_* (where *x* = [*n*(*n* + 1)(*n* + 2)]/6; *y* = [(*n* + 1)(*n* + 2)(*n* + 3)]/6). T*n* clusters have a structure corresponding to the regular tetrahedral shaped fragments of the sphalerite (cubic ZnS) phase, which is characterized by orderly distributed multivalent metal components with high tunability (Fig. 1) [2,3]. The evolution of T*n* clusters and related microcrystals has relied on modifying local charge balance, mostly by selecting the valence of the constituent metals. For example, a T2 cluster consisting of bicoordinate anionic sulfur (*μ*_2_-S^2−^) usually contains M^4+^ ions (*e.g*. Ge^4+^ or Sn^4+^) [9]. By contrast, M^3+^ ions (*e.g*. In^3+^ or Ga^3+^) can ensure the existence of tricoordinate anionic sulfur (*μ*_3_-S^2−^) to give rise to a T3 cluster [10]. However, in a T4 cluster, the tetrahedral coordination of anionic sulfur occurs within the core, which is usually stabilized by M^2+^ ions (e.g. Zn^2+^, Cd^2+^, Mn^2+^, Fe^2+^, Co^2+^ or Ni^2+^) [11]. As the cluster size increases to T5, one low-valent M^+^ ion (*e.g*. Cu^+^) located at the cluster center is connected to four *μ*_4_-S^2−^, and the 12 metal sites adjacent to the four *μ*_4_-S^2−^ are shared by high/low-valent M^3+^/M^+^ ions (*e.g*. In^3+^/Cu^+^) [12]. Furthermore, with an increasing number of *μ*_4_-S^2−^, a distinct core–subshell–shell structure emerges in the largest T6-ZnInS cluster (*i.e*. ZnS@ZnInS@InS) [13]. Larger T*n* clusters (*n* ≥ 7) are also predicted to adopt such a core–subshell–shell structure but with an increased core area, expanding toward the cubic ZnS phase. Essentially, this behavior follows Pauling's electrostatic valence rule, where the valence of an anionic sulfur should be the same as or nearly equal to the sum of its electrostatic bonds with adjacent cationic metals. Such a systematic arrangement of cationic metals endows T*n* clusters with multivalent metal components that are highly tunable, which is critical for investigating precise structure–composition–property relationships and tuning properties. Notably, high valent metal cations, such as M^3+^ or M^4+^, are not indispensable in the construction of T*n*-type clusters. It has been demonstrated that low-valent metal cations, such as Co^2+^ [14], Cd^2+^ [15] and Zn^2+^ [16], are capable of constructing T2 or T3 clusters when the surplus negative charge of anionic sulfur can be well compensated by suitable covalently protected ligands. In addition, monovalent Ag^+^ cations, which were used to build silver chalcogenide tetrahedral clusters [17,18], can also construct T3 clusters [19,20] although this was considered to be challenging because of argentophilic interactions [21].

In addition, as another excellent illustration of Pauling's electrostatic valence rule in the construction of MCSCs, the oxychalcogenide o-T*n* cluster, which is regarded as a set of pseudo-T*n* clusters, can be formed by inserting anionic oxygen (O^2−^) into the adamantane cages of the T*n* cluster and increasing the valence of the cationic metals (*i.e*. o-T2-SnOS [22], o-T3-SnOS [23] and o-T4-InSnOS [24]) to ensure local charge balance (Fig. 1). Notably, the largest o-T5 cluster dominated by In^3+^ has a unique InO_8_ core with one non-tetrahedrally coordinated In^3+^ connected to four tetrahedrally coordinated interstitial oxygens (O_i_) and four triangularly coordinated substitutional oxygens (O_s_) [25]. Interestingly, another MCSC containing non-tetrahedrally coordinated In^3+^ ions is the unique TO2 cluster, which has a NaCl-type [In_10_S_13_] core bearing octahedrally coordinated In^3+^ coupled with four [In_3_S_3_] hexagonal rings and four T2-[In_4_S_10_] clusters [26] (Fig. 1). Instead of inserting anions, the absence of core metal ions is also an effective strategy to achieve new pseudo-T*n* clusters. These coreless clusters with a central void have a single metal tetrahedral site vacant in the regular T*n* lattice, such as T5-InS (coreless) [27] and T5-CdInS (coreless) clusters [28,29]. Evidently, in both of these clusters, to compensate for the local charge mismatch, the valence of the metal coordinated to *μ*_4_-S^2−^ is increased compared with that of the metal in the T5-CuInS cluster [12]. As another type of pseudo-T*n* cluster, super-supertetrahedral T*p, q* clusters, such as T2,2-InSnS (pseudo-T4) [30], T4,2-MnInS (pseudo-T8) [31] and T3,2-InSnS (pseudo-T6) [32], are constructed by the hierarchical fusion of four T*n* clusters.

Unlike ‘naked’ polyanionic T*n*-type clusters, capped supertetrahedral C*n*-type clusters with covalently protected ligands on the surface (*e.g*. –SR or –SeR) are usually electrically neutral or have a low negative charge [2,4,5]. Regardless of the capping ligands, C*n*-type clusters have the molecular formula M*_x_*E*_y_* (where *x* = [*n*(*n* + 1)(*n* + 2)]/6 + [4(*n* + 1)(*n* + 2)]/2 + 4; *y* = [(*n* + 1)(*n* + 2)(*n* + 3)]/6 + [4(*n* + 2)(*n* + 3)]/2 + 4). Structurally, the C*n* cluster has a core possessing a regular fragment of the cubic ZnS phase covered with four corner barrelanoid cages consisting of the hexagonal *α*-ZnS phase (Fig. 1) [2,4,5]. In addition, each barrelanoid cage can be independently rotated by 60° (around the three-fold axis of the tetrahedron), resulting in four additional variants denoted as C*n, m* clusters, where *m* refers to the number of corners that have been rotated relative to the original C*n* cluster [33]. For example, the largest C*n* cluster so far is the C3,4 cluster with a composition of Cd_54_X_32_(SPh)_48_(H_2_O)_4_ (X = S, Se) [34]. Generally, the structural deformation caused by corner rotation does not affect the properties significantly; therefore, the performance is usually considered to depend on the *n* value, and Cd-17 (C1), Cd-32 (C2) and Cd-54 (C3) were usually used to emphasize the size of the cluster. When compared with the multivalent metal constituents in T*n* clusters, the employment of ER^−^ instead of E^2−^ makes charge balance less complicated and also less diverse, especially local charge balance. Therefore, C*n* clusters are usually composed of M^2+^ (*e.g.* Cd^2+^, Mn^2+^ or Hg^2+^) [4,5,35–38]. Because of the elimination of interference from multiple components, C*n* clusters are usually referred to as ultrasmall QDs for the systematic study of size-dependent optical and electronic properties.

Another series of MCSCs consists of penta-supertetrahedral P*n*-type clusters, which can be structurally considered as assemblies of four T*n* clusters capped on the face of one anti-T*n* cluster, where the anti-T*n* cluster has the same geometrical features as the T*n* cluster but with the positions of the cations and anions exchanged (Fig. 1) [2,4,5], whose configuration is very similar to the supertetrahedral zintl cluster [39]. Until now, only two types of P*n* clusters have been observed, namely, P1 and P2 clusters, each of which can be further classified by the presence or absence of covalently capped ligands. Capped P1 clusters usually have the formula [M_8_E(EPh)_16_]^2−^ (M = Zn or Cd; E = S, Se or Te) [40,41]. By contrast, ‘naked’ P1 clusters have the formula [M_4_(*μ*_4_-E)(SnE_4_)_4_]^10−^ (M = Mn, Fe, Co or Zn; E = S or Se) [42–45]. Synthetically, P2 clusters have been obtained with or without capping ligands; for example, three P2 clusters were found in 3D frameworks (P2-Li_4_In_22_S_44_ (ICF-26) [46], P2-CuInSnS (MCOF-1 and MCOF-2) [47] and P2-CuGaSnS (MCOF-4) [48]) and two discrete examples of capped P2 (P2-Cu_11_In_15_Se_16_(SePh)_24_(PPh_3_)_4_ [49] and P2-Cu(M)SnS (M = Ga, In, or both) [50]) were synthesized.

## MCSCS IN OPEN FRAMEWORKS: RETICULAR CHEMISTRY

Crystalline MCSC-based porous framework materials have attracted great attention because of the effective integration of porosity with semiconductor properties [2]. From the perspective of synthetic chemistry, the choice of sulfur provides the following advantages: (1) anionic sulfur has a larger ionic radius than oxide and fluoride ions, which favors tetrahedral coordination for cationic metals, thus allowing the formation of T*n* clusters and leading to the preparation of chalcogenide zeolite type frameworks; (2) theoretically, the higher polarizability of anionic sulfur may make M–S–M angles more flexible than the T–O–T angles in oxides, resulting in more flexible frameworks that can better accommodate a template; and (3) the arrangement of the tetrahedra in MCSCs is the same as that in bulk matter [8]. The initial synthetic methodologies for metal chalcogenide frameworks were analogous to those for oxide zeolites, with O^2−^ replaced by S^2−^ and oxyphilic metals (*e.g.* Si^4+^ or Al^3+^) replaced by sulfophilic metals (*e.g*. Ge^4+^/Sn^4+^ or Ga^3+^/In^3+^). However, the synthetic methods for metal chalcogenide frameworks have evolved over the past three decades, and current approaches can be classified as follows: (1) room- or lower-temperature solution methods (*e.g*. diffusion, evaporation or recrystallization), (2) high-temperature solid-state methods and (3) hydro(solvo)thermal or ionothermal methods. Generally, polyanionic MCSCs assemble into multilevel structures (1D chains, 2D layers or 3D frameworks) through corner sharing modes, with alkali metal ions, protonated organic amines or ionic liquids serving as structure-directing agents in addition to providing charge compensation [2,5].

**Figure 2.**
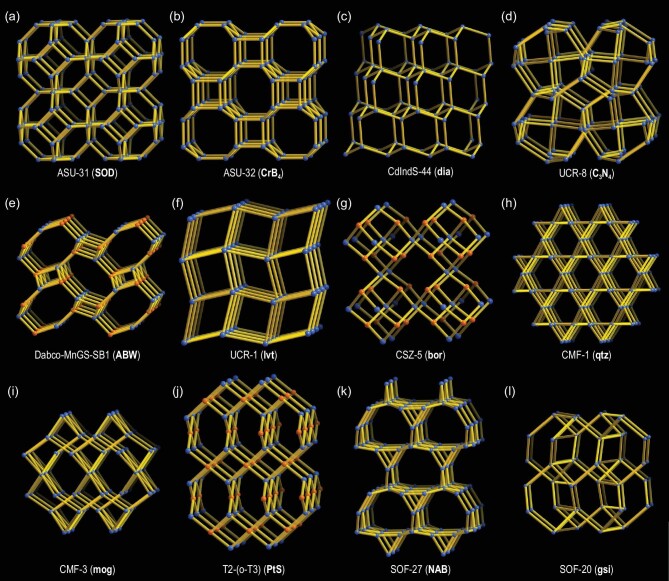
MCSC-based open frameworks with various topological structures. (a) ASU-31 with a SOD net, where T3 clusters are treated as nodes [55]. (b) ASU-32 with a CrB_4_ net, where T3 clusters are treated as nodes [55]. (c) CdInS-44 with a dia net, where T4 clusters are treated as nodes [11]. (d) UCR-8 with a cubic-C3N4 net, where *μ*_3_-S^2−^ and T4 clusters are treated as nodes [57]. (e) Dabco-MnGS-SB1 with an ABW net, where single Mn^2+^ ions and T2 clusters are treated as nodes [60]. (f) UCR-1 with an lvt net, where T4 clusters are treated as nodes [61]. (g) CSZ-5 with a bor net, where T2-InSe and interrupted T2-InSeO are treated as nodes [62]. (h) CMF-1 with a quartz (qtz) net, where P1 clusters are treated as nodes [63]. (i) CMF-3 with a mog net, where P1 and C1 clusters are treated as nodes [63]. (j) T2-(o-T3) with a PtS net, where T2 and o-T3 clusters are treated as nodes [64]. (k) SOF-27 with a NAB net, where T3 clusters are treated as nodes [65]. (l) SOF-20 with a gsi net, where T2 clusters are treated as nodes [66].

**Figure 3.**
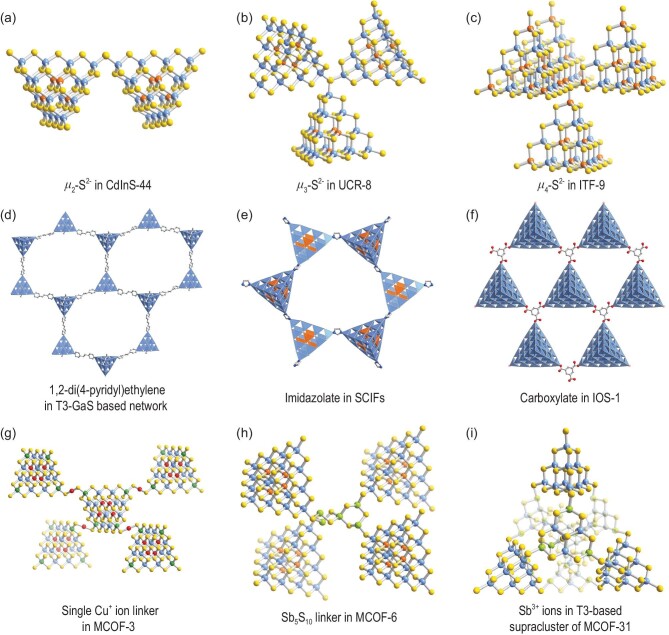
Typical sulfur connection modes in MCSC-based frameworks, exemplified by (a) *μ*_2_-S^2−^ in CdInS-44 [11], (b) *μ*_3_-S^2−^ in UCR-8 [57] and (c) *μ*_4_-S^2−^ in ITF-9 [59]. Hybrid MCSC-based frameworks: (d–f) assembly of clusters and inorganic ligands [25,76,77]. (g) Single linearly coordinated Cu^+^ ion linker in T4-CuGaSnS-based MCOF-3 [48]. (h) Sb_5_S_10_ linker in T4-MnInS-based MCOF-6 [79] and (i) Sb^3+^ ions in T3-based supracluster of MCOF-31 [80].

Research on MCSC-based open frameworks has received attention since the work by Bedard *et al.* in 1989 [51]. Early advances in the construction of such frameworks involved assembly of transition metal cations (*e.g*. Mn^2+^, Cu^+^ or Ag^+^) with T2-Ge_4_S_10_ clusters based on the efforts of the Yaghi, Ozin and Parise groups [52–54]. The first breakthrough in MCSC framework chemistry can be regarded as the formation of a framework with larger cluster size. In 1998, Parise and co-workers obtained the first T3-InS-based 3D open framework with a diamond topology, which suggested that structural variants were not limited to the Ge-S system but also possible in the In-S system [10]. Two identical subnetworks were found to exhibit undesired interpenetration. Subsequently, Yaghi and co-workers isolated three non-interpenetrated T3-InS based frameworks, including ASU-31 with a sodalite network (Fig. 2a), ASU-32 with a tetragonal CrB_4_ network (Fig. 2b) [55] and ASU-34 with a single diamond network [56]. Considering low-valent transition metals as a synthetic parameter provided a series of new open frameworks built from larger T*n* clusters, such as the first T4-CdInS-based 3D diamond-type framework CdInS-44 (Fig. 2c) [11], the first T5-CuInS-based 3D diamond-type framework UCR-17 [12] and T5-CdInS-based 2D layers [28]. Recently, the first T6-ZnInS-based 2D layered networks were also obtained [13]. Interestingly, as the cluster size increased, in addition to the common intercluster connection mode (*μ*_2_-S^2−^) (Fig. 3a), the other connection modes of S^2−^ were also developed. For example, the *μ*_3_-S^2−^ connection mode was first observed in T4-based UCR-8 (Fig. 3b) [57], affording a cubic C_3_N_4_ net with the *μ*_3_-S^2−^ and T4 clusters as nodes (Fig. 2d). The *μ*_4_-S^2−^ connection mode was first observed in T5-CuInS-based CIS-11 [58] and then in T4-ZnInS-based ITF-9 (Fig. 3c) [59]. Seemingly, the structural diversity of T*n*-based frameworks is dominated by the type of T*n* cluster (*i.e*. size and composition) and the intercluster connection modes (*i.e. μ*_2_-S^2−^, *μ*_3_-S^2−^ and *μ*_4_-S^2−^). Structure-directing agents with different charges, sizes and shapes also play an extremely important role in the framework formation, as the M–E–M bond angles can be effectively modified by the cations serving as structure-directing agents through Coulomb forces or H-bonding (H–E), leading to chalcogenide frameworks with various topologies, such as Dabco-MnGS-SB1 (ABW) [60], UCR-1 (lvt) [61], CSZ-5 (bor) [62], CMF-1 (qtz), CMF-3 (mog) [63], T2-(o-T3) (PtS) [64], SOF-27 (NAB) [65] and SOF-20 (gsi) [66] (Fig. 2e–l).

The continuous enrichment of MCSC-based frameworks and a good comprehension of synthesis methods allowed the focus to be shifted from the expansion of framework types to porosity applications. In this regard, the primary issue became effectively removing the organic templates while maintaining framework stability. Unfortunately, most of the frameworks collapsed during the ion exchange process. However, Feng and co-workers achieved a breakthrough by constructing a family of chalcogenide zeolite analogs (UCR-20–23) with excellent thermal and chemical stabilities and available void space [30]. These zeolite analogs were built from T2 clusters consisting of M^3+^/M^4+^ (*e.g.* Ge^4+^/Ga^3+^ or Sn^4+^/In^3+^) by adopting the classic stability rule of zeolite synthesis, whereby a higher M^4+^/M^3+^ ratio provides a more stable structure. Replacing M^3+^ with M^2+^ (Zn^2+^ or Cd^2+^) gave another family of high-silica-zeolite-like chalcogenides (CPM-120–123) with an M^4+^/M^2+^ ratio close to 3. Notably, CPM-120-ZnGeS exhibited reversible adsorption with high capacity and affinity for CO_2_, and could act as a robust photocatalyst [67].

Most MCSC-based frameworks have been built from clusters with the same order, which follows Pauling's fifth rule, the rule of parsimony, which states that ‘the number of essentially different kinds of constituent in a crystal tends to be small’. Nevertheless, hybrid frameworks built with different sizes or types of MCSCs are highly desirable to enrich the available structures and simultaneously realize the coexistence of multiple metal components in a single framework. In this regard, by unifying two charge-complementary synthetic strategies *via* the combination of M^2+^/M^+^, M^3+^ and M^4+^ ions, a series of hybrid MCSC-based frameworks has been successfully constructed, including T2 (GaSnS)-(o-T3) (SnOS) [64], OCF-42 (ZnGaGe(Sn)Se) built on T2–T4 clusters [68], CIS-52 built on T2 (InGeS)-T5 (CuInS) [69], UCR-19 built on T3 (GaS)-T4 (ZnGaS) [70], UCR-15 built on T3 (InS)-T5 (coreless) (InS) [27], IOS-35 built on T3(InS)-(o-T5) (InOS) [71] and OCF-45 built on T4 (MnInS)-T5 (coreless) (MnInS) [72]. In addition, efforts in the In-S (Se) or Cd-S domain have also revealed the possibility of constructing MCSC-based frameworks using hybrid P1 and T2 clusters [73,74] or hybrid P1 and C1 clusters [63]. Furthermore, by doping the In-S system with Sn^4+^ atoms to obtain clusters with appropriate global charges, a new kind of hybrid assembly between T3 and T3,2 clusters has been obtained [32].

However, following decades of development, the traditional sulfur-bridging modes have resulted in a ‘bottleneck’ in the construction of new structures. Inspired by metal–organic frameworks, the introduction of organic ligand bridges has been considered an effective approach to diversify the intercluster connection modes. Moreover, the resulting organic–inorganic hybrid framework materials may favor functional synergy. In fact, as early as 2005, bipyridine and its derivatives were introduced into C*n*-based superstructures (COV-*q*) [33,75]. By contrast, transition metal ions in C*n* clusters have a stronger coordination ability to organic ligands than the trivalent or tetravalent metals distributed at the corners of T*n* clusters. In addition, trivalent or tetravalent metals in T*n* clusters preferentially coordinate sulfur over organic ligands, especially in a reaction environment with large amounts of anionic sulfur. The third advance was realized by Vaqueiro *et al.*, who assembled T*n* clusters with 1,2-di(4-pyridyl)ethylene (Fig. 3d) [76], affording T3-GaS-based 1D and 2D covalent organic–inorganic structures. However, bipyridine ligands seem to be unwilling to form 3D framework structures with MCSCs because of their non-rigid configuration, which may make the resultant 3D framework thermodynamically unstable. Inspired by the construction of zeolitic imidazolate frameworks (ZIFs), Feng and co-workers subsequently successfully introduced imidazolate and its derivatives as linkers to form a series of 3D supertetrahedral cluster imidazolate frameworks (SCIFs), wherein the larger T4-CdInS (compared with the T3-GaS in bipyridine system) was found in SCIF-8 and SCIF-9 (Fig. 3e) [77]. In addition to N-donor ligands, carboxylate (trimesic acid), which has been widely used in the construction of metal organic frameworks (MOFs), has also been introduced as a linker in the MCSC-based structures, affording T3-InS-based 1D chain, T4-FeInS-based 0D trimer superlattice, and o-T5-InOS-based 2D honeycombed layer (Fig. 3f) [25].

As mentioned above, transition metal ions, such as Mn^2+^, Ag^+^ and Cu^+^, have been used to facilitate the assembly of MCSCs by the Yaghi, Ozin and Parise groups [52–54], but this approach is limited T2 clusters. In principle, the addition of a low-valent transition metal (M^+/2+^) to the reaction system induces the formation of larger clusters, with the transition metal present in the cluster core to stabilize the multicoordination of anionic sulfur instead of outside the cluster in a linkage. However, this issue can be addressed by adding high-valent M^4+^ (*i.e*. Ge^4+^ or Sn^4+^) to the reaction at a suitable ratio. The high-valent metals are selectively distributed at the cluster vertexes, which effectively lowers the negative charge of the terminated anionic sulfur and correspondingly reduces the possibility of further bonding of such sites to M^3+^ or M^4+^ while retaining their ability to coordinate low-valent M^+^. Based on this strategy, single Cu^+^ ion bridged MCOF-3 (Fig. 3g) and MCOF-4 (MCOF, metal chalcogenide open framework) composed of larger T4-CuGaSnS and P2-CuGaSnS clusters have been obtained [48]. Such copper-rich MCOF materials have been applied as non-enzymatic glucose-sensing catalysts and exhibit promising sensing performance. In addition to copper ions, trivalent antimony (Sb^3+^) ions have also been applied to the assembly of MCSCs, in the form of either a single Sb^3+^ ion or a small Sb*_x_*S*_y_* cluster. For example, the single Sb^3+^ cation can be incorporated into the T2-InS based framework as the tri-coordinated bridge mode [78]. Sb_5_S_10_ and Sb_6_S_12_ can, respectively, serve as tetra-coordinated linkers, giving rise to two T4-MnInS based frameworks MCOF-6 (Fig. 3h) and MOCF-7 [79]. Interestingly, because of the steric hindrance or torsion stress of those two linkers, the resultant two frameworks exhibited different symmetry of space group (*I*4_1_/a for MCOF-6 and *C*2/c for MCOF-7), further leading to the different local coordination environment of [Mn_4_S] core in T4-MnInS clusters. More recently, two new T3-based supraclusters were obtained by incorporating Sb^3+^ ions as the linker, which further make up a 2D 4,4-grid layered MCOF-31 (Fig. 3i) and a 3D pcu topological framework MCOF-32, respectively [80].

## MCSCS IN DISCRETE SUPERLATTICES: NANOCHEMISTRY

**Figure 4.**
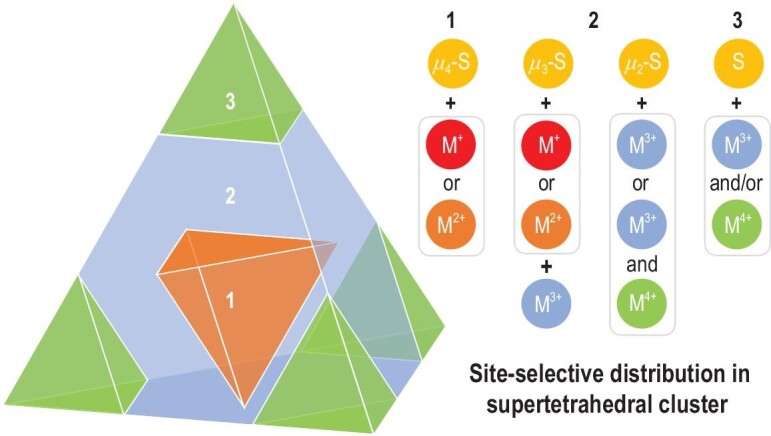
Site-selective distribution in a large T*n* supertetrahedron, where *μ*_4_-S^2−^ at the core is usually connected to low-valent M^+/2+^ ions (region 1); *μ*_3_-S^2−^ at the face is connected to M^+/2+^ and M^3+^ ions, and *μ*_2_-S^2−^ at the edge is connected to M^3+^, or M^3+^ and M^4+^ ions (region 2); and terminal S^2−^ is connected to M^3+^ and/or M^4+^ ions (region 3). Adapted with permission from [84].

Compared with MCSCs that are confined in extended frameworks, the isolated form in a superlattice provides a valuable opportunity to study the physical and chemical properties of MCSCs as genuine nanomaterials. In particular, solution-processable MCSCs are similar to II–VI or I–III–VI colloidal QDs, which can provide advantageous models for investigating various issues that are difficult to clarify using QDs. For example, a homologous series of C*n* (CdSe) clusters with well-defined electronic structures favors the systematic study of size-dependent optical and electronic properties [81], and the obtained insights may contribute to the understanding of CdSe QDs. The highly tunable multimetal components of T*n* clusters make it possible to study the photo-/electrochemical properties induced by metal components and precise doping sites and establish precise structure–composition–property relationships. Compared with the covalently capped C*n* clusters that are usually isolated in a discrete superlattice with good solution dispersity, ‘naked’ T*n* clusters, especially large ones, preferentially assemble into extended frameworks to decrease the overall negative charge of individual clusters. In principle, to obtain discrete MCSCs, the bridging ability of the anionic sulfurs at corners should be regulated to isolate the tetrahedra and the high negative charge of individual clusters should be reduced and balanced to promote successful crystallization from the mother liquor. To address these issues, a combination of ‘multivalent metal complementarity’ (or M^4+^ termination strategy) and ‘superbase-assisted crystallization’ has been proposed [3], which affords a set of discrete T4-MGaSnS clusters (M = Cu^+^, Mn^2+^ or Zn^2+^) (OCF-40) [82], a discrete T5-CuGaSnS cluster (ISC-21-CuGaSnS) [83] and three isostructural discrete P2-CuMSnS clusters (M = Ga^3+^, In^3+^ or both) [50]. To visualize the ‘multivalent metal complementarity’ strategy, an abstract representation of an isolated T4 (OCF-40) cluster is shown in Fig. 4. As mentioned previously, the metal sites with different valences are selectively distributed in the supertetrahedron to satisfy the local charge balance [84], where *μ*_4_-S^2−^ at the core is connected to low-valent M^+/2+^ ions (region 1), *μ*_3_-S^2−^ at the face is connected to M^+/2+^ and M^3+^ ions and *μ*_2_-S^2−^ at the edge is connected to M^3+^ or M^3+^ and M^4+^ ions (region 2), and terminal S^2−^ are connected to M^3+^ and/or M^4+^ ions (region 3). Notably, high-valent M^4+^ ions (usually Sn^4+^), which are typically used for preparing small T2 or T3 clusters, are introduced. The preferential distribution of these ions at the corner sites controls the bridging ability of the corner S^2−^, usually resulting in the discretization of clusters. In addition, the incorporation of a large amount of M^4+^ inevitably decreases the negative charge of the individual clusters, thus favoring crystallization and subsequent dispersion. For the ‘superbase-assisted crystallization’ strategy, superbase molecules, such as 1,8-diazabicyclo[5.4.0]undec-7-ene (DBU), 1,5-diazabicyclo[4.3.0]non-5-ene (DBN) and piperidines (PRs), which are more prone to protonation, create highly concentrated cations in the mother liquor, thus stabilizing the negatively charged MCSCs. Moreover, the approximately in-plane molecular configuration of the superbases is conducive to the crystallization of MCSCs. Compared with the control in the bridging ability of the corner S^2−^, capping four corners of MCSCs with terminated organic ligands is the most straightforward and effective method to isolate them. Although this method is very common in the C*n* and P*n* systems, it is really challenging in the T*n* system. The first success is the discrete hybrid T3-GaS (NC_7_H_9_)_4_, in which the corner S^2−^ were replaced by covalently bonded 3,5-dimethylpyridine [85]. Instead of replacing the corner S^2−^, a discrete T3-ZnGaSnSe was obtained with four corners covalently terminated by [Mn(TEPA)]^2+^ (TEPA, tetraethylenepentamine) metal complexes [86]. In addition, the DBN molecule has been experimentally demonstrated as an effective terminal agent, being similar to 3,5-dimethylpyridine, whose N atom not only has the ability to protonate but also has the ability to coordinate with cationic metals, affording a family of isolated hybrid supertetrahedral chalcogenide clusters (denoted as ISC-*n*) [87] and a unique T3-InS based dimer with six corners terminated by DBN [88,89]. More recently, imidazolium-based ionic liquids were found to be effective in preparing discrete MCSCs, and the resulting discrete superlattice exhibited good dispersibility [90].

Although an effective method has been found to address the issue of discretization, the solution dispersibility of larger T*n* clusters with a high negative charge remains challenging because of the strong electrostatic interactions caused by the close stacking of MCSCs and organic cations. In this regard, several groups have presented feasible solutions. For example, Dai and co-workers used a high ionic strength medium (Li^+^-DMF) to overcome the strong ionic forces in the crystal and realized solution-processable T5-CuInS clusters [91]. The morphology and stability of the dispersed T5-CuInS clusters were characterized by high-resolution transmission electron microscopy and electrospray ionization mass spectrometry. Our group used the principle of similar dissolution to achieve dispersion of discrete T4 clusters (OCF-40) in piperidine and obtained multimetallic sulfide nanoparticles (MMSNPs) composed of six to eight T4 clusters [92]. Similarly, the dispersion of P2-CuMSnS (M = Ga^3+^, In^3+^ or both) clusters was realized using a 1 : 1 mixture of H_2_O and acetone [50]. In addition, Li and co-workers replaced S in T4-CdInS with Se to weaken the energy of the H-bonds between T4 clusters and organic cations, thus achieving solubility in dimethyl sulfoxide (DMSO) [93]. Furthermore, the packing modes have recently been found to have an important effect on dispersibility, with T4-MInS (M = Zn^2+^ or Fe^2+^) clusters having a sodalite-net loose-packing pattern in a discrete lattice exhibiting excellent water dispersibility [94].

## PROPERTIES AND APPLICATIONS

To highlight the uniqueness and significance of these cluster-based semiconductor materials, in this section, the properties and applications of MCSC-based crystals are discussed hierarchically based on two aspects: (1) atomically precise site-dependent properties, including photo-/electrochemical properties induced by internal dopant/defect sites and external S/Se-related interfacial properties; and (2) the performance of MCSC-based crystalline semiconductor materials and functional composites.

### Atomically precise site-dependent properties

**Figure 5.**
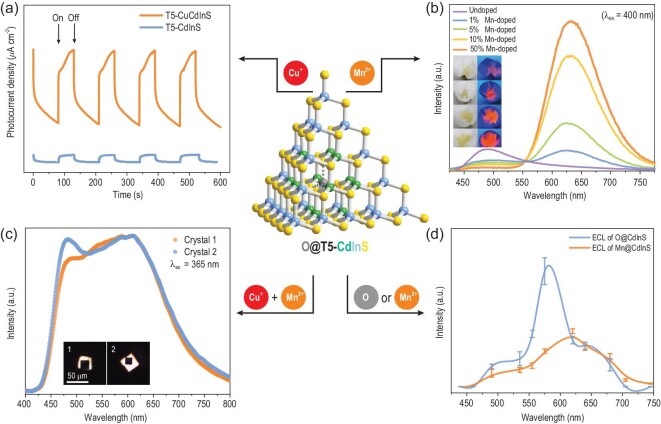
(a) Photoelectric response of T5-CdInS (coreless) and Cu-doped T5-CdInS. (b) PL of undoped and Mn-doped T5-CdInS with different doping levels (the inset shows the illuminated crystals). (c) Single-crystal white emission of Cu/Mn-codoped T5-CdInS (the inset shows the illuminated crystal). (d) ECL of T5-CdInS (coreless) and Mn-doped T5-CdInS. Adapted with permission from [29,95–97].

Doping is an effective method for tuning the electronic structures of semiconductors and their physical or chemical properties. While nanocrystals often exhibit inherent structural ambiguity and random doping sites, the atomically precise metal sites in T*n* clusters and their selective distribution provide an opportunity to gain insights into their precise structure–component–property relationships. The discrete T5-CdInS (coreless) cluster, with a single vacant metal site inside, can be doped with monometal ions. For example, the vacancy can be doped with a monocopper ion through a postmodification strategy to afford new T5-CuCdInS clusters with enhanced visible-light-responsive photoelectric properties compared with those of the parent T5-CdInS (coreless) cluster (Fig. 5a) [29]. Similarly, monomanganese doping inserted the characteristic Mn^2+^ energy levels into the T5-CdInS host, thus allowing charge or exciton energy transfer from the host lattice to the Mn-related orbitals upon excitation. This process resulted in an unusual red emission (630 nm) from the d–d spin-forbidden ^4^T_1_→^6^A_1_ transition in Mn^2+^ originating from the local ‘crystal lattice strain’ caused by the mismatch of M–S bond lengths in the ‘Mn@CdS@InS core–shell’ structure. Notably, the photoluminescence (PL) intensity is proportional to the Mn^2+^ doping level in the microcrystals composed of T5-CdInS (coreless) clusters (Fig. 5b) [95]. Furthermore, such single vacant sites can function as nanosegregation sites that eliminate possible interference between two types of dopants, giving rise to single-crystal white emission *via* the codoping of Cu^+^ and Mn^2+^ at an appropriate ratio (Fig. 5c) [96]. In addition to PL, vacancy point and antisite defects in T5-CdInS (coreless) clusters have been revealed to induce electrochemiluminescence (ECL), which can be tuned by the doped Mn^2+^ ions. As displayed in Fig. 5d, the ECL emission of the internal vacancy centered at 585 nm is suppressed when monomanganese is doped into the vacant site of T5-CdInS (coreless), which gives rise to ECL emission centered at 615 nm [97]. However, such postmodification doping strategies can lead to the following problems: (1) slow ion diffusion dynamics inevitably affect the doping level, that is, not all T5-CdInS (coreless) clusters are doped with Mn^2+^ ions; and (2) corrosion by organic solvents during the doping process may damage the host cluster and create a large number of defect points, thus providing additional non-radiative pathways. These problems are responsible for the low PL quantum yield (0.53%) [95] and low ECL efficiency (0.0085%) [97] of the postmodification Mn-doped T5-CdInS clusters. Fortunately, these problems can be addressed using the *in situ* Mn^2+^ doping method, which provides controllable Mn^2+^ doping levels and simultaneously reduces the defect points, affording a high PL quantum yield (43.68%), which is 82-fold higher than that of the sample prepared by postmodification doping [98], as well as a high ECL efficiency (27.1%) [99].

**Figure 6.**
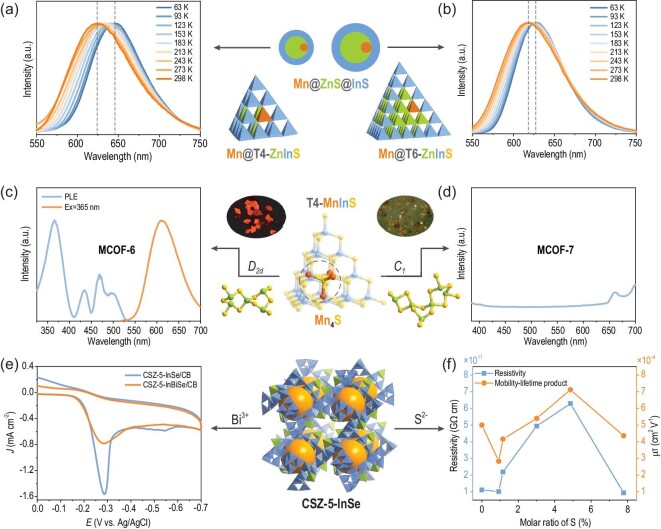
Temperature-dependent PL spectra of (a) Mn-doped T4-ZnInS and (b) Mn-doped T6-ZnInS. (c) Photoluminescence excitation (PLE) and PL spectra of MCOF-6, and (d) PL spectrum of MCOF-7. (e) CVs of ORR on CSZ-5-InSe/CB and CSZ-5-InBiSe/CB, and (f) the resistivity and mobility-lifetime product of S-doped CSZ-5-InSe versus the S doping ratio. Adapted with permission from [13,62,79,105].

It is well known that the photophysical behavior of doped semiconductors depends not only on the concentration and location of the dopants but also on the surrounding coordination environment, such as the bond length, spatial symmetry and interactions with ambient dopants [100]. Extensive research has been conducted on the former factor using colloidal QDs, whereas the latter factor can be studied using T*n* clusters. For example, doping a single Mn^2+^ ion into T4-ZnInS and T6-ZnInS yields two MnZnS@InS core–shell structures with ZnS cores of different thicknesses, in which the Mn^2+^ ions most likely replace the Zn^2+^ site on the face of the tetrahedra because of similarities in bond length. Temperature-dependent PL spectra revealed that T4-MnZnInS displays a larger red shift (∼27 nm) (Fig. 6a) than T6-MnZnInS (∼15 nm) (Fig. 6b), which is ascribed to the ‘buffering effect’ of the ZnS core, that is, a larger ZnS core can weaken the torsion or distortion of Mn–S bonds and the coordination geometry of the Mn dopant that arises from the compressive strain from the outer ‘In-S’ shell [13]. A similar phenomenon was also observed in lightly doped T4-MnZnGaSnS (MnZnS@GaSnS) and heavily doped T4-MnGaSnS (MnS@GaSnS), as the temperature-sensitive PL of the lightly doped cluster was ascribed to the larger local ‘crystal lattice strain’ caused by the non-symmetric MnZn_3_S core [101]. By contrast, in the heavily doped T4-MnInS with a Mn_4_S core, Mn–Mn magnetic coupling was expected to affect the PL behavior. Thus, the PL behavior of two T4-MnInS with different symmetrical cores (D_2d_ or C_1_) and Mn···Mn distances was investigated, revealing new insights into the dominant role of distance-directed Mn–Mn dipole–dipole interactions over symmetry-directed spin-exchange interactions in modulating the PL quenching mechanism (Fig. 6c and d) [79]. More recently, ultrafast transient spectroscopy has been employed to investigate the excited state dynamics of the internal metal sites. For example, an intercluster charger transfer process was revealed in a hybrid T3-T4 framework (UCR-19), in which the photogenerated charge carriers were directionally transferred from the T3-InS clusters to the T4-Mn(/Fe)InS clusters through the staggered band gap alignment between the molecular heterojunctions, followed by radiative/non-radiative recombination at the Mn^2+^/Fe^2+^ centers [102]. In addition, an intracluster charge transfer process was revealed in the P2-CuMInS (M = Ga, In, or both) cluster, in which three decay components in the femtosecond transient absorption spectra with systematic amplitude changes were attributed to the different constituent M^3+^ ions. Finally, a core–shell (anti-T2(CuSnS) to T2(Ga/InSnS)) charge transfer dynamic was revealed [50].

Regulation and doping of internal metal sites not only influence the photo-/electrochemical properties but also the photo-/electrocatalytic performance. For instance, a series of hybrid hydrogen evolution reaction electrocatalysts was prepared by loading MMSNPs composed of T4-MGaSnS (OCF-40) clusters on N-doped reduced graphene oxide. The Mn/Co/Zn-codoped catalyst exhibited the best electrocatalytic activity with the lowest overpotential of 176 mV at 10 mA cm^−2^ and a small Tafel slope of 43 mV dec^−1^. This result was subsequently investigated using density functional theory (DFT) calculations, which revealed that the ΔGH* of H atoms and the energy barriers for the dissociation of H atoms from H_2_O could be tuned by the doped metals, while the Mn/Co/Zn-codoped T4 cluster had a near-zero adsorption free energy for H atoms and a low dissociation barrier for H_2_O to produce adsorbed H atoms [103]. Similarly, a series of hybrid photocatalysts was prepared by coating MMSNPs on the surface of silver nanowires (Ag-NWs), in which an ultrathin Ag_2_S interface layer formed to act as an adhesive between the MMSNPs and the Ag-NWs. This system exhibited tunable visible-light photocatalytic performance through the synergistic effect of the multimetallic constituents of the MMSNPs [92]. In addition to the transition metals, the interrupted In^3+^ sites in the T2-InSeO cluster of the CSZ-5-InSe framework were demonstrated to be electrocatalytically active centers for the oxygen reduction reaction (ORR); the electro-/photoelectrochemical performance was easily manipulated by replacing In^3+^ at interrupted sites with Bi^3+^, and the cyclic voltammograms (CVs) for the ORR on CSZ-5-InSe/CB and CSZ-5-InBiSe/CB show that Bi^3+^ doping at interrupted sites can deteriorate the catalytic activity [62] (Fig. 6e).

In addition to the metal component, the chalcogen elements can also be modified to effectively tune the electronic structure of MCSC-based crystals and their properties. For example, as mentioned above, replacement of S^2−^ with Se^2−^ can promote the dispersibility of T4 clusters. Moreover, subsequent photocatalytic experiments showed that the H_2_ evolution rate increases significantly as the Se content in the T4 cluster increases [93]. Another interesting case is the nonlinear variation in the composition and optical band gap of CSZ-5-InSe as the Se^2−^ sites are gradually replaced with S^2−^, which results from the structural features of this open framework [104]. In addition, the nonlinear control mechanism of CSZ-5 could result in an optimal balance between resistivity, band gap and carrier mobility, thus affording an excellent X-ray detector with a high figure of merit for the mobility–lifetime product (7.12 × 10^−4^ cm^2^ V^−1^) [105] (Fig. 6f).

Because of the soft Lewis base nature of chalcogen elements (S, Se, and Te), the polarizability of the internal surface of MCSC-based frameworks is much larger than that of oxide zeolites and porous carbons [106]. In addition, the highly negative charge density of MCSC-based frameworks can afford a high cation uptake capacity, with the multidimensional intersecting channels allowing rapid ion diffusion and offering a unique kinetic advantage. These features are highly desirable for ion exchange and gas adsorption. For example, UCR-20 exhibited a highly selective and rapid uptake of radionuclide Cs^+^ after activation through a stepwise ion-exchange strategy [107], and the activated UCR-20 also showed highly selective adsorption of CO_2_ over N_2_ [108]. Moreover, an investigation of the Cs^+^ exchange kinetics in T4-InSnOS-based framework materials by Zhang and co-workers found that the small pore pockets created by the two interpenetrating frameworks act as pincers to selectively capture Cs^+^ ions [109]. In addition, a series of purely inorganic MCSC-based frameworks, which was prepared *in situ* using alkali cation templates, exhibited high ionic conductivities (up to 1.8 × 10^−2^ Ω^−1^ cm^−1^) at room temperature and moderate to high humidity because of their high anionic framework polarizability and high concentrations of mobile alkali metal cations [110].

### Cluster and/or framework-dependent functional properties

**Figure 7.**
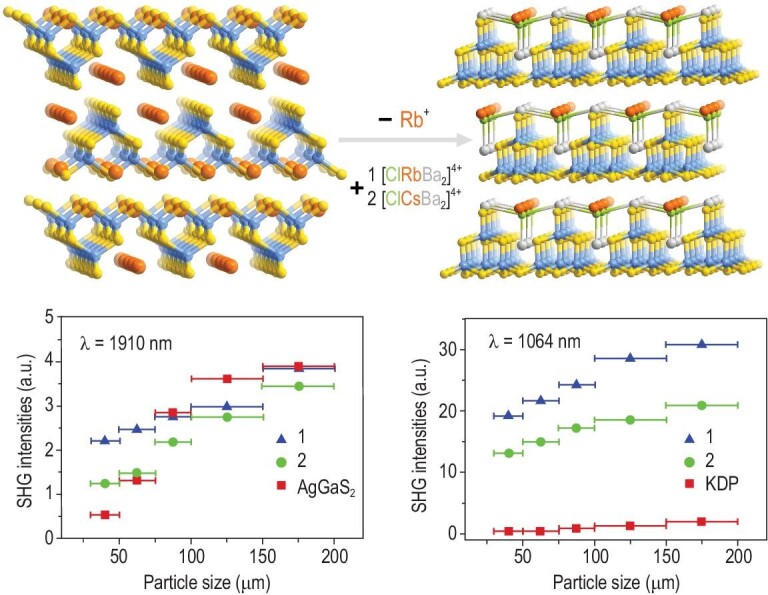
Polycation-substitution-induced centrosymmetric transformation of T2-based materials and particle-size-dependent SHG intensities of T2-based crystals with incident lasers at 1910 and 1064 nm, respectively. Adapted with permission from [121].

In addition to the abovementioned site-dependent properties, MCSCs can also exhibit cluster and/or framework-dependent properties. Thus, this section focuses on the properties and applications of MCSC-based crystalline semiconductor materials and MCSC-based functional composites. Generally, MCSC-based framework materials have been investigated as photocatalysts for hydrogen generation from water [67,111], dye degradation [112] and the reduction of CO_2_ into CH_4_ [113]. Furthermore, electrocatalysis applications, mainly focusing on the ORR [62,114,115], continue to be reported. In addition to these conventional applications, some unique properties inherent to MCSCs have also been explored. For example, the intrinsic advantages of the integrated porosity and semiconductor properties make MCSC-based frameworks a good model for investigating host–guest chemistry. For instance, by encapsulating acridine orange (AO) in the nanopores and loading rhodamine B (RhB) on the surface of the semiconductor porous framework UCR-20, respectively, a multistep vectorial host-guest energy transfer can be clearly observed, in which the ultraviolet energy harvested by the host framework can transfer to the first-order acceptor AO molecules, then on to the second-order acceptor RhB molecules, resulting in visible light emission [116]. Similarly, a series of host–guest synergetic electrocatalysts was fabricated by embedding Cu_2_S [117] or S-doped Ni(OH)_2_ [118] nanoparticles in the nanopores of UCR-20. Generally, the host framework not only plays an important role in improving the electrocatalytic performance of the composite but also acts as a sulfur source and stabilizer. As a result of their good aqueous dispersibility, as mentioned above, discrete T2 clusters have been used as precursors to construct crystalline porous frameworks. These clusters can also be used as precursors to prepare amorphous porous semiconductor materials, such as chalcogenide aerogels, and Kanatzidis and co-workers have made a great contribution in this area [119]. Typically, T2 clusters (GeS, GeSe or SnSe) are proportionally mixed with K_2_[PtCl_4_] in aqueous solution, resulting in all the Cl ligands being replaced with the terminal S or Se atoms of T2 clusters, which eventually affords gelation [120]. The non-centrosymmetry of the tetrahedral T*n* clusters is a prerequisite for the generation of nonlinear optical (NLO) materials, especially for second harmonic generation (SHG). For example, using acentric [ABa_2_Cl] polycations (A = Rb or Cs) to replace Rb^+^ cations in parent centrosymmetric RbGaS_2_, two new non-centrosymmetric salt-inclusion chalcogenides [ABa_2_Cl][Ga_4_S_8_] with ordered arrangement of NLO-active T2-Ga_4_S_10_ clusters were achieved, which demonstrated strong phase-matchable SHG intensities, high laser-induced damage thresholds, and a wide transparency window [121] (Fig. 7).

## CONCLUSION AND OUTLOOK

In conclusion, the development of MCSC chemistry over the past three decades has centered on the expansion of MCSCs (size, type and composition) and MCSC-based frameworks, and exploration of their properties and applications. Essentially, development of MCSCs is inseparable from development of MCSC-based frameworks and *vice versa*. Although it is undeniable that more elaborate structures can still be achieved through the assembly of MCSCs using traditional sulfur connection modes, new synthesis strategies are also essential. To date, only a few types of organic ligands have been successfully introduced into MCSC-based frameworks. Therefore, from the perspective of developing new structures and realizing organic–inorganic synergy materials, the exploration of organic–inorganic hybrid frameworks should be extended. In regard to the discretization and dispersibility of MCSCs, various effective strategies have been presented and promising results have been achieved. However, the dispersion of clusters in solution, especially monodispersion, remains a significant challenge that must be addressed for postmodification and subsequent applications. In addition to the materials covered in this review, MCSCs have also been demonstrated to form surfactant-encapsulated complexes (SECs) with quaternary ammonium salts bearing long carbon chains. These SECs are promising precursors for the amorphous self-assembly of MCSCs. In addition, MCSCs are promising triplet sensitizers for photon upconversion or reactive oxygen species generation, for which MCSC composition and site-dependent regulatory mechanisms are likely to be significant.

## References

[1] Dehnen S . Clusters-Contemporary Insight in Structure and Bonding. Cham: Springer, 2017.

[2] Feng P , Bu X, Zheng N. The interface chemistry between chalcogenide clusters and open framework chalcogenides. Acc Chem Res 2005; 38: 293–303. 10.1021/ar0401754 15835876

[3] Zhang J , Bu X, Feng P et al. Metal chalcogenide supertetrahedral clusters: synthetic control over assembly, dispersibility, and their functional applications. Acc Chem Res 2020; 53: 2261–72. 10.1021/acs.accounts.0c00381 32877164

[4] Levchenko TI , Huang Y, Corrigan JF. Large metal chalcogenide clusters and their ordered superstructures via solvothermal and ionothermal syntheses. In: Dehnen S (ed.). Clusters-Contemporary Insight in Structure and Bonding. Cham: Springer, 2017, 269–320.

[5] Vaqueiro P. Hybrid materials through linkage of chalcogenide tetrahedral clusters. Dalton Trans 2010; 39: 5965–72. 10.1039/c000130a 20396808

[6] Flanigen EM. Zeolites and molecular sieves: an historical perspective. In: van Bekkum H, Flanigen EM, Jacobs PA et al. (eds.). Studies in Surface Science and Catalysis. Amsterdam: Elsevier, 2001, 11–35.

[7] van Koningsveld H. How to build zeolites. In: van Bekkum H, Flanigen EM, Jacobs PA et al. (eds.). Studies in Surface Science and Catalysis. Amsterdam: Elsevier, 2001, 69–173.

[8] Férey G. Supertetrahedra in sulfides: matter against mathematical series? Angew Chem Int Ed 2003; 42: 2576–9. 10.1002/anie.200201621 12813732

[9] Philippot E , Ribs M, Lindqvist O. Crystal structure of Na_4_Ge_4_S_10_. Rev Chim Miner 1971; 8: 477–89. 10.1021/ic034659s

[10] Cahill CL , Ko Y, Parise JB. A novel 3-dimensional open framework sulfide based upon the [In_10_S_20_]^10−^ supertetrahedron: DMA-InS-SB1. Chem Mater 1998; 10: 19–21. 10.1021/cm9705707

[11] Li H , Kim J, Groy TL et al. 20 Å Cd_4_In_16_S_35_^14−^ supertetrahedral T4 clusters as building units in decorated cristobalite frameworks. J Am Chem Soc 2001; 123: 4867–8. 10.1021/ja010413f 11457310

[12] Bu X , Zheng N, Li Y et al. Pushing up the size limit of chalcogenide supertetrahedral clusters: two- and three-dimensional photoluminescent open frameworks from (Cu_5_In_30_S_54_)^13−^ clusters. J Am Chem Soc 2002; 124: 12646–7. 10.1021/ja021009z 12392396

[13] Xu X , Wang W, Liu D et al. Pushing up the size limit of metal chalcogenide supertetrahedral nanocluster. J Am Chem Soc 2018; 140: 888–91. 10.1021/jacs.7b12092 29337544

[14] Dance IG , Calabrese JC. X-ray structure of the hexa(μ_2_-arenethiolato-)tetra(arenethiolato)tetracobaltate(II) dianion, [(CoSPh)_4_(μ_2_-SPh)_6_]^2^^−^, a new tetrahedral tetracobalt thiolate cluster. J Chem Soc Chem Commun 1975: 762–3. 10.1039/C39750000762

[15] Dance IG , Garbutt RG, Craig DC et al. The different nonmolecular polyadamantanoid crystal structures of cadmium benzenethiolate and 4-methylbenzenethiolate. Analogies with microporous aluminosilicate frameworks. Inorg Chem 1987; 26: 4057–64. 10.1021/ic00271a019

[16] Dance IG , Choy A, Scudder ML. Syntheses, properties, and molecular and crystal structures of (Me_4_N)_4_[E_4_M_10_(SPh)_16_] (E = sulfur or selenium; m = zinc or cadmium): molecular supertetrahedral fragments of the cubic metal chalcogenide lattice. J Am Chem Soc 1984; 106: 6285–95. 10.1021/ja00333a030

[17] Wang Z , Liu J-W, Su H-F et al. Chalcogens-induced Ag_6_Z_4_@Ag_36_ (Z = S or Se) core-shell nanoclusters: enlarged tetrahedral core and homochiral crystallization. J Am Chem Soc 2019; 141: 17884–90. 10.1021/jacs.9b09460 31602974

[18] Li X-Y , Su H-F, Yu K et al. A platonic solid templating Archimedean solid: an unprecedented nanometre-sized Ag_37_ cluster. Nanoscale 2015; 7: 8284–8. 10.1039/C5NR01222H 25882899

[19] Luo G-G , Su H-F, Xiao A et al. Silver-sulfur hybrid supertetrahedral clusters: the hitherto missing members in the metal-chalcogenide tetrahedral clusters. Chem Eur J 2017; 23: 14420–4. 10.1002/chem.201703468 28875580

[20] Xu C , Hedin N, Shi H-T et al. A semiconducting microporous framework of Cd_6_Ag_4_(SPh)_16_ clusters interlinked using rigid and conjugated bipyridines. Chem Commun 2014; 50: 3710–2. 10.1039/C3CC49660K 24577529

[21] Schmidbaur H , Schier A. Argentophilic interactions. Angew Chem Int Ed 2015; 54: 746–84. 10.1002/anie.201405936 25393553

[22] Ahari H , Lough A, Petrov S et al. Modular assembly and phase study of two- and three-dimensional porous tin(IV) selenides. J Mater Chem 1999; 9: 1263–74. 10.1039/a807660j

[23] Schiwy W , Krebs B. Sn_10_O_4_S^8^^−^_20_: a new type of polyanion. Angew Chem Int Ed 1975; 14: 436. 10.1002/anie.197504361

[24] Zhang X-M , Sarma D, Wu Y-Q et al. Open-framework oxysulfide based on the supertetrahedral [In_4_Sn_16_O_10_S_34_]^12^^−^ cluster and efficient sequestration of heavy metals. J Am Chem Soc 2016; 138: 5543–6. 10.1021/jacs.6b02959 27082786

[25] Yang H , Zhang J, Luo M et al. The largest supertetrahedral oxychalcogenide nanocluster and its unique assembly. J Am Chem Soc 2018; 140: 11189–92. 10.1021/jacs.8b07349 30088766

[26] Wu T , Zuo F, Wang L et al. A large indium sulfide supertetrahedral cluster built from integration of ZnS-like tetrahedral shell with NaCl-like octahedral core. J Am Chem Soc 2011; 133: 15886–9. 10.1021/ja2066994 21923195

[27] Wang C , Bu X, Zheng N et al. Nanocluster with one missing core atom: a three-dimensional hybrid superlattice built from dual-sized supertetrahedral clusters. J Am Chem Soc 2002; 124: 10268–9. 10.1021/ja020735z 12197715

[28] Su W , Huang X, Li J et al. Crystal of semiconducting quantum dots built on covalently bonded T5 [In_28_Cd_6_S_54_]^−12^: the largest supertetrahedral cluster in solid state. J Am Chem Soc 2002; 124: 12944–5. 10.1021/ja027830s 12405810

[29] Wu T , Zhang Q, Hou Y et al. Monocopper doping in Cd-In-S supertetrahedral nanocluster via two-step strategy and enhanced photoelectric response. J Am Chem Soc 2013; 135: 10250–3. 10.1021/ja404181c 23819843

[30] Zheng N , Bu X, Wang B et al. Microporous and photoluminescent chalcogenide zeolite analogs. Science 2002; 298: 2366–9. 10.1126/science.1078663 12493908

[31] Li H , Kim J, O’Keeffe M et al. [Cd_16_In_64_S_134_]^44^^−^: 31-Å tetrahedron with a large cavity. Angew Chem Int Ed 2003; 42: 1819–21. 10.1002/anie.200250748 12722070

[32] Wang W , Yang H, Xue C et al. The first observation on dual self-closed and extended assembly modes in supertetrahedral T3 cluster based open-framework chalcogenide. Cryst Growth Des 2017; 17: 2936–40. 10.1021/acs.cgd.7b00403

[33] Zheng N , Bu X, Lu H et al. One-dimensional assembly of chalcogenide nanoclusters with bifunctional covalent linkers. J Am Chem Soc 2005; 127: 14990–1. 10.1021/ja055376x 16248614

[34] Zheng N , Bu X, Lu H. Crystalline superlattices from single-sized quantum dots. J Am Chem Soc 2005; 127: 11963–5. 10.1021/ja053588o 16117534

[35] Lee GSH , Craig DC, Ma I et al. [S_4_Cd_17_(SPh)_28_]^2−^, the first member of a third series of tetrahedral [SWMX(SR)y]z- clusters. J Am Chem Soc 1988; 110: 4863–4. 10.1021/ja00222a075

[36] Herron N , Calabrese JC, Farneth WE et al. Crystal structure and optical properties of Cd_32_S_14_(SC_6_H_5_)_36__·_ DMF4, a cluster with a 15 angstrom CdS core. Science 1993; 259: 1426–8. 10.1126/science.259.5100.1426 17801274

[37] Eichhöfer A , Wood PT, Viswanath RN et al. Synthesis, structure and physical properties of the manganese(ii) selenide/selenolate cluster complexes [Mn_32_Se_14_(SePh)_36_(P*n*Pr_3_)_4_] and [Na(benzene-15-crown-5)(C_4_H_8_O)_2_]_2_[Mn_8_Se(SePh)_16_]. Chem Commun 2008; 1596–8. 10.1039/b714582a 18354811

[38] Behrens S , Bettenhausen M, Deveson AC et al. Synthesis and structure of the Nanoclusters [Hg_32_Se_14_(SePh)_36_], [Cd_32_Se_14_(SePh)_36_-(PPh_3_)_4_], [P(Et)_2_(Ph)C_4_H_8_OSiMe_3_]_5_-[Cd_18_I_17_(PSiMe_3_)_12_], and [N(Et)_3_C_4_H_8_OSiMe_3_]_5_[Cd_18_I_17_(PSiMe_3_)_12_]. Angew Chem Int Ed 1996; 35: 2215–8. 10.1002/anie.199622151

[39] Xu H-L , Popov IA, Tkachenko NV et al. σ-Aromaticity-induced stabilization of heterometallic supertetrahedral clusters [Zn_6_Ge_16_]^4^^−^ and [Cd_6_Ge_16_]^4^^−^. Angew Chem Int Ed 2020; 59: 17286–90. 10.1002/anie.202008276 32608037

[40] Dance IG. [ClZn_8_(SPh)_16_]^−^, a new, highly symmetrical cage containing metal atoms inside and outside an icosahedron of ligands; X-ray crystallographic study. J Chem Soc Chem Commun 1980; 818–20. 10.1039/C39800000818

[41] Lee GSH , Fisher KJ, Craig DC et al. [ECd_8_(E’Ph)_16_]^2−^ cluster chemistry (E, E' = sulfur, selenium, tellurium). J Am Chem Soc 1990; 112: 6435–7. 10.1021/ja00173a063

[42] Zimmermann C , Melullis M, Dehnen S. Reactivity of chalcogenostannate salts: unusual synthesis and structure of a compound containing ternary cluster anions [Co_4_(μ_4_-Se)(SnSe_4_)_4_]^10–^. Angew Chem Int Ed 2002; 41: 4269–72. 10.1002/1521-3773(20021115)41:22<4269::AID-ANIE4269>3.0.CO;2-8 12434359

[43] Dehnen S , Brandmayer MK. Reactivity of chalcogenostannate compounds: syntheses, crystal structures, and electronic properties of novel compounds containing discrete ternary anions [M^II^_4_(μ_4_-Se)(SnSe_4_)_4_]^10−^ (M^II^= Zn, Mn). J Am Chem Soc 2003; 125: 6618–9. 10.1021/ja029601b 12769556

[44] Palchik O , Iyer RG, Liao JH et al. K_10_M_4_Sn_4_S_17_ (M = Mn, Fe, Co, Zn): soluble quaternary sulfides with the discrete [M_4_Sn_4_S_17_]^10−^ supertetrahedral clusters. Inorg Chem 2003; 42: 5052–4. 10.1021/ic034600l 12924876

[45] Palchik O , Iyer RG, Canlas CG et al. K_10_M_4_M’_4_S_17_ (M = Mn, Fe, Co, Zn; M’= Sn, Ge) and Cs_10_Cd_4_Sn_4_S_17_: compounds with a discrete supertetrahedral cluster. Z Anorg Allg Chem 2004; 630: 2237–47. 10.1002/zaac.200400154

[46] Zheng NF , Bu XH, Feng PY. Pentasupertetrahedral clusters as building blocks for a three-dimensional sulfide superlattice. Angew Chem Int Ed 2004; 43: 4753–5. 10.1002/anie.200460386 15366077

[47] Lv J , Zhang J, Xue C et al. Two penta-supertetrahedral cluster-based chalcogenide open frameworks: effect of the cluster spatial connectivity on the electron-transport efficiency. Inorg Chem 2019; 58: 3582–5. 10.1021/acs.inorgchem.8b03503 30793596

[48] Zhang J , Wang X, Lv J et al. A multivalent mixed-metal strategy for single-Cu^+^-ion-bridged cluster-based chalcogenide open frameworks for sensitive nonenzymatic detection of glucose. Chem Commun 2019; 55: 6357–60. 10.1039/C9CC02905B 31065633

[49] Eichhöfer A , Fenske D. Syntheses and structures of new copper(I)-indium(III)-selenide clusters. J Chem Soc Dalton Trans 2000; 6: 941–4. 10.1039/a909737f

[50] Zhang J , Qin C, Zhong Y et al. Atomically precise metal-chalcogenide semiconductor molecular nanoclusters with high dispersibility: designed synthesis and intracluster photocarrier dynamics. Nano Res 2020; 13: 2828–36. 10.1007/s12274-020-2936-0

[51] Bedard RL , Wilson ST, Vail LD et al. The next generation: synthesis, characterization, and structure of metal sulfide-based microporous solids. In: Jacobs PA, van Santen RA (eds.). Studies in Surface Science and Catalysis. Amsterdam: Elsevier, 1989, 375–87.

[52] Yaghi OM , Sun Z, Richardson DA et al. Directed transformation of molecules to solids: synthesis of a microporous sulfide from molecular germanium sulfide cages. J Am Chem Soc 1994; 116: 807–8. 10.1021/ja00081a067

[53] Tan K , Darovsky A, Parise JB. Synthesis of a novel open-framework sulfide, CuGe_2_S_5_·(C_2_H_5_)_4_N, and its structure solution using synchrotron imaging plate data. J Am Chem Soc 1995; 117: 7039–40. 10.1021/ja00131a042

[54] Bowes CL , Huynh WU, Kirkby SJ et al. Dimetal linked open frameworks: [(CH_3_)_4_N]_2_(Ag_2_, Cu_2_)Ge_4_S_10_. Chem Mater 1996; 8: 2147–52. 10.1021/cm960280a

[55] Li H , Laine A, O’Keeffe M et al. Supertetrahedral sulfide crystals with giant cavities and channels. Science 1999; 283: 1145–7. 10.1126/science.283.5405.1145 10024236

[56] Li H , Eddaoudi M, Laine A et al. Noninterpenetrating indium sulfide supertetrahedral cristobalite framework. J Am Chem Soc 1999; 121: 6096–7. 10.1021/ja990410r

[57] Bu X , Zheng N, Li Y et al. Templated assembly of sulfide nanoclusters into cubic-C_3_N_4_ type framework. J Am Chem Soc 2003; 125: 6024–5. 10.1021/ja030103s 12785810

[58] Wang L , Wu T, Zuo F et al. Assembly of supertetrahedral T5 copper-indium sulfide clusters into a super-supertetrahedron of infinite order. J Am Chem Soc 2010; 132: 3283–5. 10.1021/ja9100672 20178361

[59] Zhang L , Xue C, Wang W et al. Stable supersupertetrahedron with infinite order via the assembly of supertetrahedral T4 zinc-indium sulfide clusters. Inorg Chem 2018; 57: 10485–8. 10.1021/acs.inorgchem.8b01233 30118223

[60] Cahill CL , Parise JB. Synthesis and structure of MnGe_4_S_10_·(C_6_H_14_N_2_)·3H_2_O: a novel sulfide framework analogous to zeolite Li-A(BW). Chem Mater 1997; 9: 807–11. 10.1021/cm960484r

[61] Wang C , Li Y, Bu X et al. Three-dimensional superlattices built from (M4In16S33)10- (M = Mn, Co, Zn, Cd) supertetrahedral clusters. J Am Chem Soc 2001; 123: 11506–7. 10.1021/ja011739r 11707140

[62] Lin J , Dong Y, Zhang Q et al. Interrupted chalcogenide-based zeolite-analogue semiconductor: atomically precise doping for tunable electro-/photoelectrochemical properties. Angew Chem Int Ed 2015; 54: 5103–7. 10.1002/anie.201500659 25727727

[63] Zhang Q , Bu X, Zhang J et al. Chiral semiconductor frameworks from cadmium sulfide clusters. J Am Chem Soc 2007; 129: 8412–3. 10.1021/ja072274t 17567135

[64] Han X , Wang Z, Liu D et al. Co-assembly of a three-dimensional open framework sulfide with a novel linkage between an oxygen-encapsulated T3 cluster and a supertetrahedral T2 cluster. Chem Commun 2014; 50: 796–8. 10.1039/C3CC45439H 24292374

[65] Wang W , Wang X, Hu D et al. An unusual metal chalcogenide zeolitic framework built from the extended spiro-5 units with supertetrahedral clusters as nodes. Inorg Chem 2018; 57: 921–5. 10.1021/acs.inorgchem.7b03057 29308887

[66] Wu Z , Wang X-L, Hu D et al. A new cluster-based chalcogenide zeolite analogue with a large inter-cluster bridging angle. Inorg Chem Front 2019; 6: 3063–9. 10.1039/C9QI01051C

[67] Lin Q , Bu X, Mao C et al. Mimicking high-silica zeolites: highly stable germanium- and tin-rich zeolite-type chalcogenides. J Am Chem Soc 2015; 137: 6184–7. 10.1021/jacs.5b03550 25950820

[68] Wu T , Wang X, Bu X et al. Synthetic control of selenide supertetrahedral clusters and three-dimensional co-assembly by charge-complementary metal cations. Angew Chem Int Ed 2009; 48: 7204–7. 10.1002/anie.200903758 19718736

[69] Wang L , Wu T, Bu X et al. Coassembly between the largest and smallest metal chalcogenide supertetrahedral clusters. Inorg Chem 2013; 52: 2259–61. 10.1021/ic301965w 23421915

[70] Zheng N , Bu X, Feng P. Nonaqueous synthesis and selective crystallization of gallium sulfide clusters into three-dimensional photoluminescent superlattices. J Am Chem Soc 2003; 125: 1138–9. 10.1021/ja021274k 12553794

[71] Zhang J , Liu X, Ding Y et al. Three new metal chalcogenide open frameworks built through co-assembly and/or hybrid assembly from supertetrahedral T5-InOS and T3-InS nanoclusters. Dalton Trans 2019; 48: 7537–40. 10.1039/C9DT01410A 31066399

[72] Wang H , Wang W, Hu D et al. Hybrid assembly of different-sized supertetrahedral clusters into a unique non-interpenetrated Mn-In-S open framework with large cavity. Inorg Chem 2018; 57: 6710–5. 10.1021/acs.inorgchem.8b00907 29792414

[73] Zhang Q , Bu X, Han L et al. Two-dimensional indium sulfide framework constructed from pentasupertetrahedral P1 and supertetrahedral T2 clusters. Inorg Chem 2006; 45: 6684–7. 10.1021/ic060367q 16903723

[74] Xue C , Hu D, Zhang Y et al. Two unique crystalline semiconductor zeolite analogues based on indium selenide clusters. Inorg Chem 2017; 56: 14763–6. 10.1021/acs.inorgchem.7b02718 29199823

[75] Zheng N , Bu X, Lauda J et al. Zero- and two-dimensional organization of tetrahedral cadmium chalcogenide clusters with bifunctional covalent linkers. Chem Mater 2006; 18: 4307–11. 10.1021/cm060557z

[76] Vaqueiro P , Romero ML. Gallium-sulfide supertetrahedral clusters as building blocks of covalent organic-inorganic networks. J Am Chem Soc 2008; 130: 9630–1. 10.1021/ja801619e 18597468

[77] Wu T , Khazhakyan R, Wang L et al. Three-dimensional covalent co-assembly between inorganic supertetrahedral clusters and imidazolates. Angew Chem Int Ed 2011; 50: 2536–9. 10.1002/anie.201006531 21370332

[78] Wang K-Y , Feng M-L, Li J-R et al. [NH_3_CH_3_]_4_[In_4_SbS_9_SH]: a novel methylamine-directed indium thioantimonate with Rb^+^ ion-exchange property. J Mater Chem A 2013; 1: 1709–15. 10.1039/C2TA00710J

[79] Liu Y , Zhang J, Han B et al. New insights into Mn-Mn coupling interaction-directed photoluminescence quenching mechanism in Mn^2+^-doped semiconductors. J Am Chem Soc 2020; 142: 6649–60. 10.1021/jacs.0c00156 32176486

[80] Ding Y , Zhang J, Liu C et al. Antimony-assisted assembly of basic supertetrahedral clusters into heterometallic chalcogenide supraclusters. Inorg Chem 2020; 59: 13000–4. 10.1021/acs.inorgchem.0c02097 32886495

[81] Soloviev VN , Eichhöfer A, Fenske D et al. Size-dependent optical spectroscopy of a homologous series of CdSe cluster molecules. J Am Chem Soc 2001; 123: 2354–64. 10.1021/ja003598j 11456885

[82] Wu T , Wang L, Bu X et al. Largest molecular clusters in the supertetrahedral Tn series. J Am Chem Soc 2010; 132: 10823–31. 10.1021/ja102688p 20681716

[83] Hu R , Wang X-L, Zhang J et al. Multi-metal nanocluster assisted Cu-Ga-Sn tri-doping for enhanced photoelectrochemical water splitting of BiVO_4_ film. Adv Mater Interfaces 2020; 7: 2000016. 10.1002/admi.202000016

[84] Wu T , Bu X, Zhao X et al. Phase selection and site-selective distribution by tin and sulfur in supertetrahedral zinc gallium selenides. J Am Chem Soc 2011; 133: 9616–25. 10.1021/ja203143q 21595469

[85] Vaqueiro P , Romero ML. [Ga_10_S_16_(NC_7_H_9_)_4_]^2−^: a hybrid supertetrahedral nanocluster. Chem Commun 2007: 3282–4. 10.1039/b704724j 17668101

[86] Xu G , Guo P, Song S et al. Molecular nanocluster with a [Sn_4_Ga_4_Zn_2_Se_20_]^8^^−^ T3 supertetrahedral core. Inorg Chem 2009; 48: 4628–30. 10.1021/ic900376h 19466797

[87] Wu T , Bu X, Liao P et al. Superbase route to supertetrahedral chalcogenide clusters. J Am Chem Soc 2012; 134: 3619–22. 10.1021/ja210039u 22335388

[88] Sun L , Zhang H-Y, Zhang J et al. A quasi-D3-symmetrical metal chalcogenide cluster constructed by the corner-sharing of two T3 supertetrahedra. Dalton Trans 2020; 49: 13958–61. 10.1039/D0DT02420A 33021307

[89] Wu J , Jin B, Wang X et al. Breakdown of valence shell electron pair repulsion theory in an H-bond-stabilized linear sp-hybridized sulfur. CCS Chem 2020; 2: 2584–90. 10.31635/ccschem.020.202000471

[90] Peng Y , Hu Q, Liu Y et al. Discrete supertetrahedral Tn chalcogenido clusters synthesized in ionic liquids: crystal structures and photocatalytic activity. ChemPlusChem 2020; 85: 2487–98. 10.1002/cplu.202000639 33215856

[91] Li Z-Q , Mo C-J, Guo Y et al. Discrete supertetrahedral CuInS nanoclusters and their application in fabrication of cluster-sensitized TiO_2_ photoelectrodes. J Mater Chem A 2017; 5: 8519–25. 10.1039/C7TA00247E

[92] Liu D , Liu Y, Huang P et al. Highly tunable heterojunctions from multimetallic sulfide nanoparticles and silver nanowires. Angew Chem Int Ed 2018; 57: 5374–8. 10.1002/anie.201800848 29655189

[93] Hao M , Hu Q, Zhang Y et al. Soluble supertetrahedral chalcogenido T4 clusters: high stability and enhanced hydrogen evolution activities. Inorg Chem 2019; 58: 5126–33. 10.1021/acs.inorgchem.9b00207 30946583

[94] Xue C , Zhang L, Wang X et al. Enhanced water dispersibility of discrete chalcogenide nanoclusters with a sodalite-net loose-packing pattern in a crystal lattice. Inorg Chem 2020; 59: 15587–94. 10.1021/acs.inorgchem.0c00621 32410454

[95] Lin J , Zhang Q, Wang L et al. Atomically precise doping of monomanganese ion into coreless supertetrahedral chalcogenide nanocluster inducing unusual red shift in Mn^2+^ emission. J Am Chem Soc 2014; 136: 4769–79. 10.1021/ja501288x 24625310

[96] Lin J , Wang L, Zhang Q et al. Highly effective nanosegregation of dual dopants in a micron-sized nanocluster-based semiconductor molecular single crystal for targeting white-light emission. J Mater Chem C 2016; 4: 1645–50. 10.1039/C5TC04191K

[97] Wang F , Lin J, Zhao T et al. Intrinsic-vacancy point defect-induced electrochemiluminescence from coreless supertetrahedral chalcogenide nanocluster. J Am Chem Soc 2016; 138: 7718–24. 10.1021/jacs.6b03662 27228563

[98] Lin J , Hu D-D, Zhang Q et al. Improving photoluminescence emission efficiency of nanocluster-based materials by in situ doping synthetic strategy. J Phys Chem C 2016; 120: 29390–6. 10.1021/acs.jpcc.6b09126

[99] Wang F , Lin J, Yu S et al. Anti-Site defects-assisted enhancement of electrogenerated chemiluminescence from in situ Mn^2+^-doped supertetrahedral chalcogenide nanoclusters. ACS Appl Mater Interfaces 2018; 10: 38223–9. 10.1021/acsami.8b13635 30362345

[100] Yang X , Pu C, Qin H et al. Temperature- and Mn^2+^ concentration-dependent emission properties of Mn^2+^-doped ZnSe nanocrystals. J Am Chem Soc 2019; 141: 2288–98. 10.1021/jacs.8b08480 30649864

[101] Zhang Q , Lin J, Yang Y-T et al. Exploring Mn^2+^-location-dependent red emission from (Mn/Zn)-Ga-Sn-S supertetrahedral nanoclusters with relatively precise dopant positions. J Mater Chem C 2016; 4: 10435–44. 10.1039/C6TC03844A

[102] Xue C , Fan X, Zhang J et al. Direct observation of charge transfer between molecular heterojunctions based on inorganic semiconductor clusters. Chem Sci 2020; 11: 4085–96. 10.1039/D0SC00458H 34122874 PMC8152627

[103] Liu D , Fan X, Wang X et al. Cooperativity by multi-metals confined in supertetrahedral sulfide nanoclusters to enhance electrocatalytic hydrogen evolution. Chem Mater 2019; 31: 553–9. 10.1021/acs.chemmater.8b04665

[104] Lin J , Hu D, Yang H et al. Nonlinear variation in the composition and optical band gap of an alloyed cluster-based open-framework metal chalcogenide. Inorg Chem 2018; 57: 4248–51. 10.1021/acs.inorgchem.8b00542 29611702

[105] Wu S , Liang C, Zhang J et al. A photoconductive X-ray detector with a high figure of merit based on an open-framework chalcogenide semiconductor. Angew Chem Int Ed 2020; 59: 18605–10. 10.1002/anie.202010290 32777154

[106] Manos MJ , Kanatzidis MG. Metal sulfide ion exchangers: superior sorbents for the capture of toxic and nuclear waste-related metal ions. Chem Sci 2016; 7: 4804–24. 10.1039/C6SC01039C 30155129 PMC6016724

[107] Yang H , Luo M, Luo L et al. Highly selective and rapid uptake of radionuclide cesium based on robust zeolitic chalcogenide via stepwise ion-exchange strategy. Chem Mater 2016; 28: 8774–80. 10.1021/acs.chemmater.6b04273

[108] Yang H , Luo M, Chen X et al. Cation-exchanged zeolitic chalcogenides for CO_2_ adsorption. Inorg Chem 2017; 56: 14999–5005. 10.1021/acs.inorgchem.7b02307 29192766

[109] Wang L , Pei H, Sarma D et al. Highly selective radioactive ^137^Cs^+^ capture in an open-framework oxysulfide based on supertetrahedral cluster. Chem Mater 2019; 31: 1628–34. 10.1021/acs.chemmater.8b04877

[110] Zheng N , Bu X, Feng P. Synthetic design of crystalline inorganic chalcogenides exhibiting fast-ion conductivity. Nature 2003; 426: 428–32. 10.1038/nature02159 14647378

[111] Zheng N , Bu X, Vu H et al. Open-framework chalcogenides as visible-light photocatalysts for hydrogen generation from water. Angew Chem Int Ed 2005; 44: 5299–303. 10.1002/anie.200500346 16038010

[112] Nie L , Zhang Q. Recent progress in crystalline metal chalcogenides as efficient photocatalysts for organic pollutant degradation. Inorg Chem Front 2017; 4: 1953–62. 10.1039/C7QI00651A

[113] Sasan K , Lin Q, Mao C et al. Open framework metal chalcogenides as efficient photocatalysts for reduction of CO_2_ into renewable hydrocarbon fuel. Nanoscale 2016; 8: 10913–6. 10.1039/C6NR02525K 27186825

[114] Zhang Y , Hu D, Xue C et al. A 3D neutral chalcogenide framework built from a supertetrahedral T3 cluster and a metal complex for the electrocatalytic oxygen reduction reaction. Dalton Trans 2018; 47: 3227–30. 10.1039/C7DT04891B 29423493

[115] Wang W , Wang X, Zhang J et al. Three-dimensional superlattices based on unusual chalcogenide supertetrahedral In-Sn-S nanoclusters. Inorg Chem 2019; 58: 31–4. 10.1021/acs.inorgchem.8b02574 30550271

[116] Hu D-D , Lin J, Zhang Q et al. Multi-step host-guest energy transfer between inorganic chalcogenide-based semiconductor zeolite material and organic dye molecules. Chem Mater 2015; 27: 4099–104. 10.1021/acs.chemmater.5b01158

[117] Hu D , Wang X, Yang H et al. Host-guest electrocatalyst with cage-confined cuprous sulfide nanoparticles in etched chalcogenide semiconductor zeolite for highly efficient oxygen reduction reaction. Electrochim Acta 2018; 282: 877–85. 10.1016/j.electacta.2018.06.106

[118] Hu D , Wang X, Chen X et al. S-Doped Ni(OH)_2_ nano-electrocatalyst confined in semiconductor zeolite with enhanced oxygen evolution activity. J Mater Chem A 2020; 8: 11255–60. 10.1039/D0TA00547A

[119] Bag S , Arachchigea IU, Kanatzidis MG. Aerogels from metal chalcogenides and their emerging unique properties. J Mater Chem 2008; 18: 3628–32. 10.1039/b804011g

[120] Bag S , Trikalitis PN, Chupas PJ et al. Porous semiconducting gels and aerogels from chalcogenide clusters. Science 2007; 317: 490–3. 10.1126/science.1142535 17656718

[121] Liu B-W , Jiang X-M, Zeng H-Y et al. [ABa_2_Cl][Ga_4_S_8_] (A = Rb, Cs): wide-spectrum nonlinear optical materials obtained by polycation-substitution-induced nonlinear optical (NLO)-functional motif ordering. J Am Chem Soc 2020; 142: 10641–5. 10.1021/jacs.0c04738 32469217

